# Cyclophosphamide Chemotherapy Sensitizes Tumor Cells to TRAIL-Dependent CD8 T Cell-Mediated Immune Attack Resulting in Suppression of Tumor Growth

**DOI:** 10.1371/journal.pone.0006982

**Published:** 2009-09-10

**Authors:** Robbert G. van der Most, Andrew J. Currie, Amanda L. Cleaver, Joanne Salmons, Anna K. Nowak, Sathish Mahendran, Irma Larma, Amy Prosser, Bruce W. S. Robinson, Mark J. Smyth, Anthony A. Scalzo, Mariapia A. Degli-Esposti, Richard A. Lake

**Affiliations:** 1 National Research Centre for Asbestos Related Diseases, University of Western Australia, Perth, Australia; 2 School of Medicine and Pharmacology, University of Western Australia, Perth, Australia; 3 Cancer Immunology Program, Sir Donald and Lady Trescowthick Laboratories, Peter MacCallum Cancer Centre, East Melbourne, Australia; 4 Centre for Experimental Immunology, Lions Eye Institute, and Immunology and Virology Program, Centre for Ophthalmology and Visual Science, The University of Western Australia, Nedlands, Australia; New York University School of Medicine, United States of America

## Abstract

**Background:**

Anti-cancer chemotherapy can be simultaneously lymphodepleting and immunostimulatory. Pre-clinical models clearly demonstrate that chemotherapy can synergize with immunotherapy, raising the question how the immune system can be mobilized to generate anti-tumor immune responses in the context of chemotherapy.

**Methods and Findings:**

We used a mouse model of malignant mesothelioma, AB1-HA, to investigate T cell-dependent tumor resolution after chemotherapy. Established AB1-HA tumors were cured by a single dose of cyclophosphamide in a CD8 T cell- and NK cell-dependent manner. This treatment was associated with an IFN-α/β response and a profound negative impact on the anti-tumor and total CD8 T cell responses. Despite this negative effect, CD8 T cells were essential for curative responses. The important effector molecules used by the anti-tumor immune response included IFN-γ and TRAIL. The importance of TRAIL was supported by experiments in nude mice where the lack of functional T cells could be compensated by agonistic anti-TRAIL-receptor (DR5) antibodies.

**Conclusion:**

The data support a model in which chemotherapy sensitizes tumor cells for T cell-, and possibly NK cell-, mediated apoptosis. A key role of tumor cell sensitization to immune attack is supported by the role of TRAIL in tumor resolution and explains the paradox of successful CD8 T cell-dependent anti-tumor responses in the absence of CD8 T cell expansion.

## Introduction

Tumor antigens are cross-presented to the immune system [1], [2], [3]. However, the ensuing anti-tumor CD8 T cell response is usually not effective and fails to control tumor growth. Indeed, the immunological outcome of antigen cross-presentation is determined by the context in which tumor antigens are presented. Altering that context is an important goal for anti-cancer immunotherapy [4], [5], [6]. Cytotoxic chemotherapy can play a role in this process since apoptotic tumor cell death can be an immunostimulatory event (‘immunogenic cell death’) [7], [8], [9], potentially adding an immunostimulatory signal to cross-presented antigens. An immune priming effect has now been shown for several chemotherapeutic drugs, including gemcitabine [4], [10] and doxorubicin [7]. As a result, chemo- and immunotherapy are no longer considered to be *a priori* antagonistic [11] and the concept of combined chemo-immuno therapy is receiving more attention [11], [12], [13]. However, the notion that chemotherapy and anti-tumor T cell responses can be synergistic must be reconciled with the fact that many chemotherapeutic drugs deplete lymphocytes [14]. In fact, lymphodepletion after chemotherapy was the main reason why chemo- and immunotherapy were seen as antagonistic. The growing insight that chemotherapy can be immunostimulatory presents a paradox: how are effective anti-tumor T cell responses generated under lymphodepleting conditions? The present study aims to address the paradoxical relationship between immunogenicity and lymphodepletion. To study this, we have used a mouse model of malignant mesothelioma (AB1-HA), which is sensitive to both chemotherapy and immunotherapy [4], [15], [16], in combination with the chemotherapeutic drug cyclophosphamide (CY), since CY treatment is associated with innate and adaptive immune activation [17], [18], [19], [20], [21]. The AB1-HA tumor cell line was generated by transfection of the asbestos-induced AB1 tumor cell line [16] with the influenza virus HA gene [15]. The tumor-expressed HA protein allows us to monitor the anti-tumor T cell response [5], [15], [22], but it does not impact on tumor immunogenicity, as evidenced by the fact that AB1-HA cured mice are also protected against re-challenge with the parental AB1 line [23].

The immuno-stimulatory properties of CY (Cytoxan™) have been known for decades. In the 1980s, it was shown that CY depleted cycling suppressor T cells, now known as regulatory T cells, and thereby activated anti-tumor CD8 T cells [24]. However, we have recently shown that the anti-tumor efficacy of CY in the AB1-HA model cannot be explained by regulatory T cell depletion alone [18]. Here, we show that CY kills tumor cells by apoptosis and that it has a CD8 T cell- and NK cell-dependent anti-tumor effect in the AB1-HA tumor model. At the same time, CY has a strong negative effect on T cell proliferation, limiting the potential expansion of anti-tumor CD8 T cells, raising the question how a CD8 T cell-dependent anti-tumor response functions without T cell expansion. We found that the anti-tumor immune response depended on different effector molecules to eliminate the tumor: IFN-γ and TRAIL. The role of TRAIL was supported by data showing that agonistic anti-TRAIL-receptor (DR5) antibodies enhanced the effects of CY in athymic nude mice. Thus, a DR5-agonist compensates for the lack of functional T cell in nude mice. The role of TRAIL suggests that the efficacy of CY can be explained by tumor cell sensitization for T cell and/or NK cell apoptosis. A model in which CY sensitizes tumor cells for TRAIL-mediated death may help explain the chemotherapy paradox, since such a model emphasizes tumor cell susceptibility rather than expansion of anti-tumor CD8 T cells.

## Results

The objective of this study was to investigate the potential discrepancy between lymphodepleting anti-tumor chemotherapy and the anti-tumor immune responses that are linked to successful chemotherapy [11], [25]. To test this, we used a murine mesothelioma model (AB1-HA) that is sensitive to both chemotherapy and immunotherapy [1], [4], [18], in combination with the DNA cross-linker cyclophosphamide (CY).

### Apoptotic cell death after maphosphamide treatment of tumor cells

**Figure 1:**
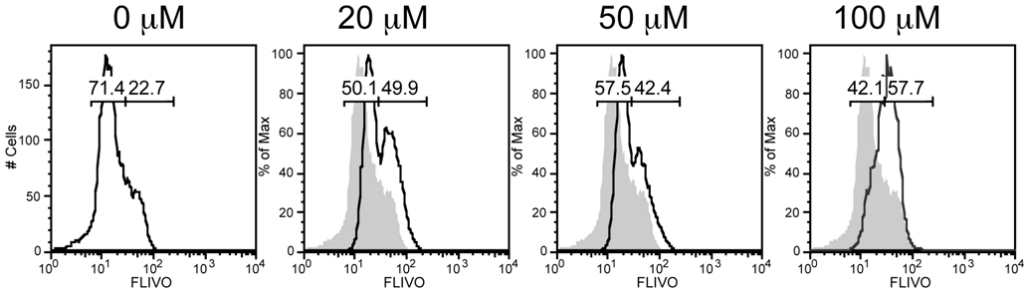
The CY metabolite maphosphamide kills by inducing apoptosis. AB1-HA tumor cells were treated with different doses of maphosphamide for 24 h and stained with FLIVO reagent.

As we have recently shown [18], AB1-HA murine mesothelioma cells are killed by maphosphamide, which is the active metabolite of CY. To further determine how CY kills tumor cells, AB1-HA cells were treated with the active metabolite maphosphamide at different concentrations for 24 hours. Induction of apoptosis was evaluated using the fluorescent FAM-VAD-fmk pan-caspase staining reagent (FLIVO), which binds activated caspases [26]. I*n vitro* exposure of tumor cells to maphosphamide increased the proportion of FAM-VAD-fmk reactive cells in a dose-dependent manner, suggesting that cell death was associated with pan-caspase activation and hence apoptosis (**Figure 1**). Similarly, FAM-VAD-fmk staining cells were observed after *in vivo* treatment of tumor-bearing mice with cyclophosphamide (**data not shown**). Thus, as we show here, the CY metabolite maphosphamide induces apoptosis in AB1-HA tumor cells, providing a plausible explanation for the *in vitro* cytotoxic effect of maphosphamide and the *in vivo* anti-tumor effect of CY in athymic nude mice, as previously demonstrated [18].

### Immunogenicity of CY chemotherapy

**Figure 2:**
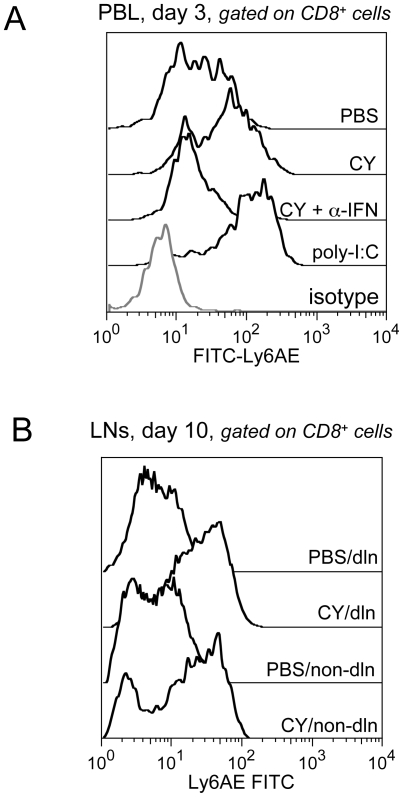
Type-I IFN production assessed by Ly6AE upregulation on CD8 T cells. Data shown are representative histograms displaying the shift in staining intensity from isotype control to Ly6AE^hi^ CD8 T cells from one of three tumor-bearing mice per treatment group. Poly(I:C) was used as a positive control for Ly6AE expression. (A) From top to bottom: Ly6AE on CD8 T cells in PBS-treated mice, CY-treated mice, CY + anti-IFN-α/β treated mice, poly-I:C treated mice and isotype control staining (tinted curve), in PBL samples, day 3 after i.p. CY injection. (B) Type-I IFN responses are long-lived and systemic. Ly6AE expression in tumor draining and non-draining lymph nodes analyzed at day 10 post CY injection. CY-treated mice and untreated mice are compared.

Apoptotic tumor cell death can be immune-stimulatory [7]. Thus, to evaluate the immune effects of CY-induced tumor cell apoptosis, we analyzed changes in gene expression within CY-treated tumors using real time PCR SuperArrays, allowing simultaneous analysis of 84 cytokine genes using real-time PCR. Preliminary data indicate that several pro-inflammatory genes were upregulated in CY-treated tumors, including type-I IFNs (IFN-α4 and IFN-β1) but also IL-1α, IL-19, IL-2 and OX40L (Van der Most, unpublished data). Type-I IFN production after CY treatment was further analyzed by measuring the expression levels of Ly6AE on CD8 T cells. Ly6AE upregulation on T cells is a very early non-antigen-specific (bystander) response of T cells to IFN-exposure [27], [28] and can therefore serve as a surrogate for type-I IFN (IFN-α/β) production. Indeed, we have recently confirmed that Ly6AE-upregulation on CD8 T cells occurs in a strictly IFN-α/β-dependent manner after AB1-HA tumor treatment with the TLR3 and TLR7 agonists poly-I:C and imiquimod [5], [23]. Therefore, to assess IFN-α/β production, we measured Ly6AE expression on peripheral blood CD8 T cells harvested three days after administration of CY (150 mg/kg) in AB1-HA tumor-bearing mice. We found that CY treatment resulted in an upregulation of Ly6AE expression on the majority of CD8 T cells in the PBL pool when compared to untreated tumor-bearing mice (**Figure 2A**), consistent with systemic exposure of the CD8 T cells to IFN-α/β. The level of upregulation was similar to that observed with (intratumoral) poly-I:C injection (**Figure 2A**). Neutralization of type-I IFNs, using an anti-IFN-α/β serum, completely inhibited Ly6AE upregulation on peripheral blood CD8 T cells (**Figure 2A**), confirming our previous finding that upregulation of Ly6AE expression is a *bona fide* marker for systemic type-I IFN release [5], [23]. To evaluate the longevity of the IFN-α/β response, we analyzed Ly6AE expression on day 10 after CY injection in the tumor draining and non-draining lymph nodes. The results clearly show sustained Ly6AE upregulation on CD8 T cells in both draining and non-draining lymph nodes (**Figure 2B**), demonstrating that CY injection results in long-lasting and systemic type-I IFN release. Therefore, the combined data suggest that CY-injection in tumor-bearing mice results in systemic immune activation, consistent with the concept of immunogenic cell death [7], [18] and consistent with previously reported data [17], [29].

### Anti-tumor responses depend on CD8 T cells but not on IFN-αβ

**Figure 3:**
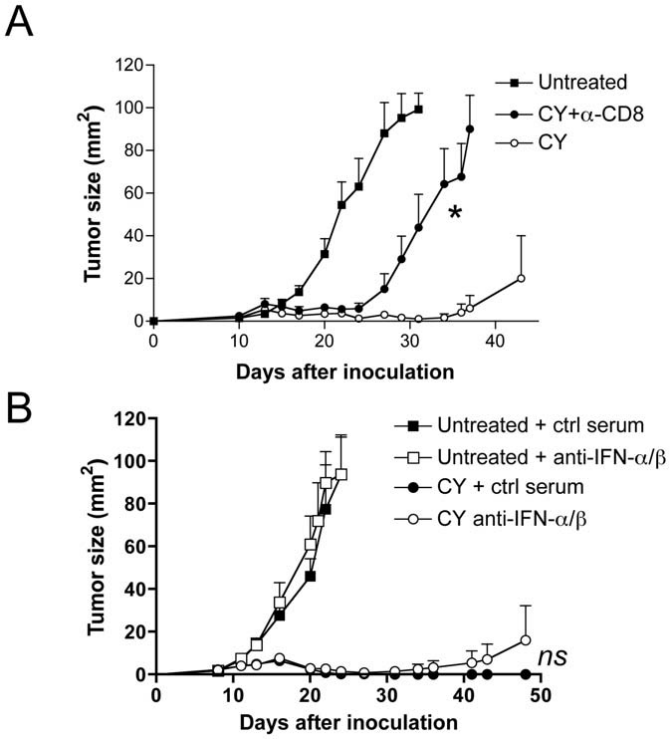
Role of CD8 T cells and type-I IFN in CY-driven anti-tumor efficacy. (A) Anti-tumor responses depend on CD8^+^ T cells. Tumor-bearing mice were treated with CY at day 10 after tumor inoculation (day 0) and anti-CD8 mAbs (150 µg) were injected at days −1, 0, 2, 4, 6 and 8 with respect to CY treatment. Data shown are mean±SEM (*n* = 5) from one representative experiment from a total of three experiments with a total of 15 mice. * P<0.05 when CY is compared with CY + anti-CD8. (B) *In vivo* IFN-α/β neutralization marginally affects tumor growth in CY-treated mice. BALB/c mice bearing AB1-HA tumors were given IFN-α/β blocking antibody on days −1, +2, +4 with respect to CY treatment. Tumors were inoculated at day 0 and were treated with CY at day 9. Data shown are mean±SEM (*n* = 5) from one experiment. *ns* = not-significant.

CY has a strong CD8 T cell-dependent anti-tumor effect in AB1-HA tumor-bearing mice, routinely leading to 75–100% cure rate when small (i.e., just palpable) tumors are treated (**Figure 3A**), confirming our previous data [18], whereas CD8 depletion alone had a weak effect accelarating tumor growth (P = 0.07, data not shown). As we demonstrated previously, CY treatment in tumor-bearing athymic nude mice (which lack functional T cells) only had a transient anti-tumor effect and did not result in any curative anti-tumor responses [18]. Thus, T cells play an essential role in achieving cures. Since CY treatment is associated with an IFN-α/β response and because primary CD8 T cell responses to apoptotic cells and cross-presented antigens are type-I IFN-dependent [8], [30], we hypothesized that the type-I IFN response could be responsible for priming the anti-tumor CD8 T cell response. This was tested by *in vivo* neutralization of IFN-α/β. We found that the anti-tumor efficacy of CY was only marginally affected by type-I IFN neutralization (**Figure 3B**), indicating that the CY-induced anti-tumor CD8 T cell response was not dependent on IFN-α/β. As a positive control for the efficiency of in vivo IFN-α/β-neutralization, we have shown that poly-I:C-mediated anti-tumor responses were abrogated using this anti-IFN-α/β treatment [5]. Thus, our data suggest that the CY-induced anti-tumor T cell response is type-I IFN independent and may therefore not be a *de novo* response because such a response would be type-I IFN-dependent [8]. Instead, we reasoned that CY could make existing responses more effective, either by expanding tumor-specific CD8 T cells or by increasing the sensitivity of tumor cells to T cell-mediated apoptosis. To test this, we further studied anti-tumor CD8 T cell responses after CY treatment.

### Impaired CD8 T cell responses after CY treatment

**Figure 4:**
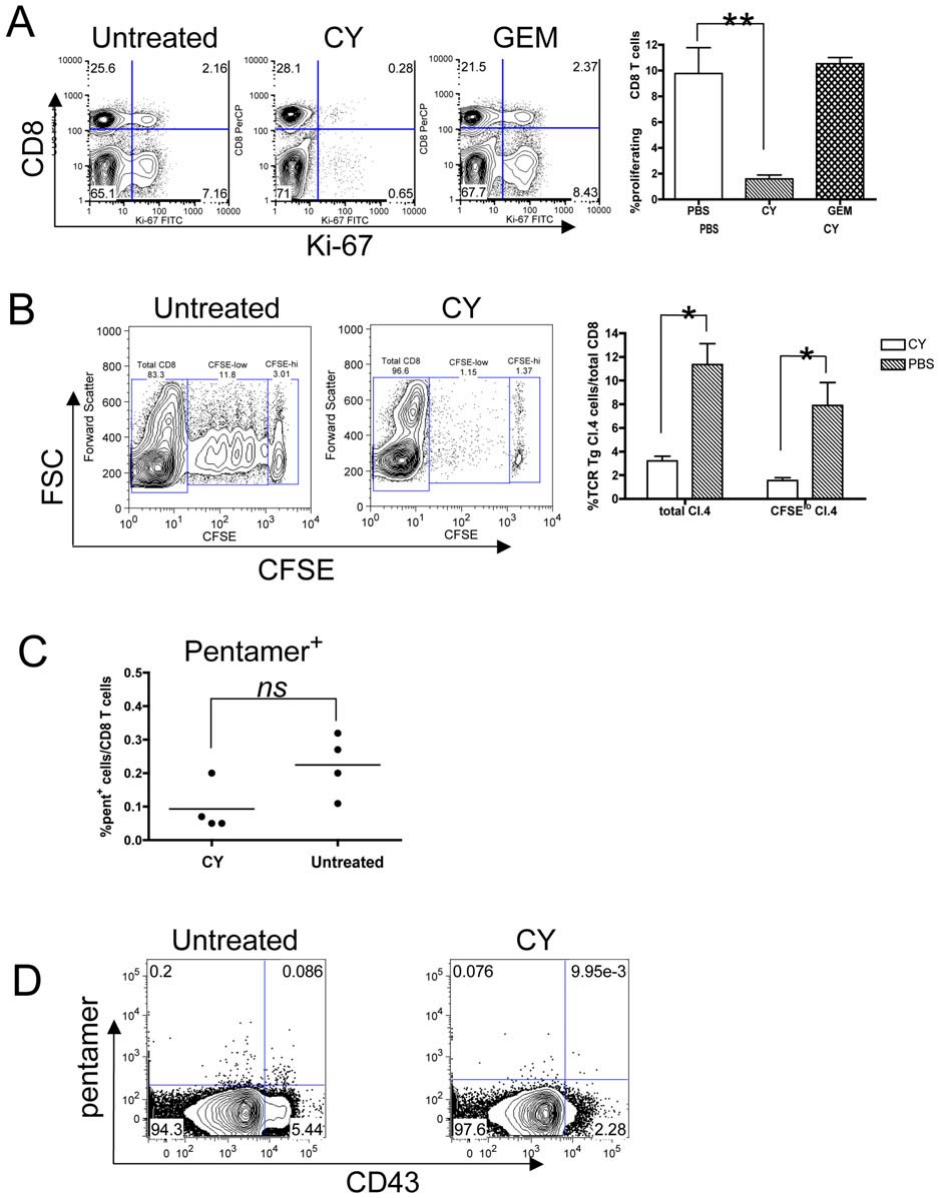
Impaired proliferation of CD8 T cells after CY treatment. (A) Draining lymph node cells from tumor-bearing mice treated with saline, CY or gemcitabine (GEM) at day 10 post inoculation were analyzed for Ki-67 expression three days later. Cells shown were gated such that CD8^neg^ cells are CD4 T cells. Data shown in lower panel are mean±SEM (*n* = 3) from one experiment. ** P<0.01 when PBS is compared with CY (unpaired t test). (B) Adoptively transferred transgenic CD8 T cells fail to proliferate in CY-treated mice. CFSE-labeled HA-specific clone 4 cells were transferred into tumor bearing mice two days before PBS or CY injection and were harvested 7 days later and stained for CD8 expression. Cells shown are gated as CD8^+^. Data shown in lower panel are mean±SEM (*n* = 3) from one experiment. * P<0.05 when PBS is compared with CY (unpaired t test). (C) HA-specific MHC class I pentamer staining in untreated and CY-treated tumor-bearing mice. Data shown are frequencies of pentamer+ CD8 T cells per total CD8 T cells in tumor-draining lymph nodes at day 10 post CY injection. *ns* = not significant (P = 0.06, unpaired t test). (D) CD43 (clone 1B11) expression on pentamer^+^ CD8 T cells in tumor-draining lymph nodes. Cells shown are gated on CD8^+^ cells. Representative plots are shown.

CY is a cytotoxic and lymphodepleting drug. A single dose of CY (150 mg/kg) resulted in a 75% reduction of splenic cellularity [18] as well as of total CD8 T cell numbers (**data not shown**) Thus, expansion of anti-tumor CD8 T cells after CY chemotherapy seems paradoxical. To study this, we characterized total and tumor-specific CD8 T cell responses after CY treatment, using three different experimental approaches. First, we assessed CD8 T cell proliferation in the tumor-draining lymph nodes after CY treatment by intracellular Ki-67 staining. Ki-67 is a nuclear marker that is upregulated in cycling cells [18], [31], [32]. Interestingly, CY treatment selectively depleted cycling cells, as shown by the almost complete absence of Ki-67^hi^ CD8 T cells three days after a single CY injection (**Figure 4A**). We have recently reported similar findings for CD4 T cells and foxp3^+^ CD4^+^ regulatory T cells [18]. This specific loss of Ki-67^hi^ T cells was not seen with a different cytotoxic drug, gemcitabine (GEM) (**Figure 4A**), despite the fact that GEM causes lymphodepletion in mice [14]. Since both drugs are cytotoxic, the difference between CY and GEM may be one of kinetics, in which the CY-mediated T cell proliferation arrest is more long-lived. Second, to assess to the impact of CY on the tumor-specific CD8 T cell response, we adoptively transferred TCR-transgenic CD8 T cells into CY treated mice. Clone 4 transgenic CD8 T cells recognize a K^d^-restricted epitope from the influenza virus HA protein which is expressed as a tumor *neo*-antigen in AB1-HA cells [15]. Ten million CFSE-labeled tumor-specific transgenic CD8 T cells were adoptively transferred into mice at day 7 after tumor cell inoculation. Mice were treated with CY (150 mg/kg) at day 9 and lymphocytes were isolated from the tumor-draining and non-draining lymph nodes at day 14 (i.e., 5 days after CY injection and 7 days after adoptive transfer). Confirming previous work employing this same assay [1], we observed robust proliferation of tumor-specific CD8 T cells in the tumor-draining lymph nodes of untreated control tumor-bearing mice, as can be inferred from the serial dilution of CFSE dye intensity (**Figure 4B**). However, both the recovery of tumor-specific transgenic T cells as well as proliferation of these cells (assessed by serial CFSE dilution) were significantly reduced in the draining lymph nodes from CY-treated mice (**Figure 4B**). These findings are consistent with the notion that CY has a negative effect on T cell proliferation. Third, to further define the specificity of CD8 T cell responses after CY treatment we used a MHC class I pentamer specific for the K^d^-restricted HA epitope to visualize the endogenous response to the HA epitope. Thus, these experiments were done without adoptive T cell transfer. Since CY-induced lymphopenia is transient, with splenic cellularity recovering within 10 days after treatment (**data not shown**), we reasoned that early inhibition of T cell proliferation could be followed by recovery of the anti-tumor CD8 T cell response. Therefore, we measured CD8 T cell responses at day 10 after CY injection. Low frequencies of pentamer-positive HA-specific CD8 T cells were measured in the draining lymph nodes of both untreated and CY-treated tumor-bearing mice (**Figure 4C**) and there were no significant differences, although there was a trend towards lower frequencies of pentamer-positive cells after CY treatment (P = 0.06). Phenotypic characterization of pentamer+ cells using the cytotoxicity marker CD43 [33] revealed that in untreated tumor-bearing mice, both CD43^hi^ and CD43^lo^ pentamer+ tumor specific CD8 T cells were present (**Figure 4D**), whereas the small populations of pentamer+ CD8 T cells in CY-treated mice appeared to be CD43^lo^. Overall, these data suggests that these T cells do not possess a highly activated and cytotoxic phenotype. However, despite this lack of CD8 T cell expansion, we found that mice that were cured through CY treatment invariably resisted rechallenge with AB1-HA tumor cells (n = 20, **data not shown**), which indicates that the CD8 T cell response that was mobilized by CY treatment eventually generated tumor-specific memory.

In conclusion, the combined data shown here reveal a discrepancy between the key role of CD8 T cells in tumor resolution and the strong anti-proliferative affects of CY on CD8 T cells.

### Antitumor responses depend on IFN-γ and perforin

**Figure 5:**
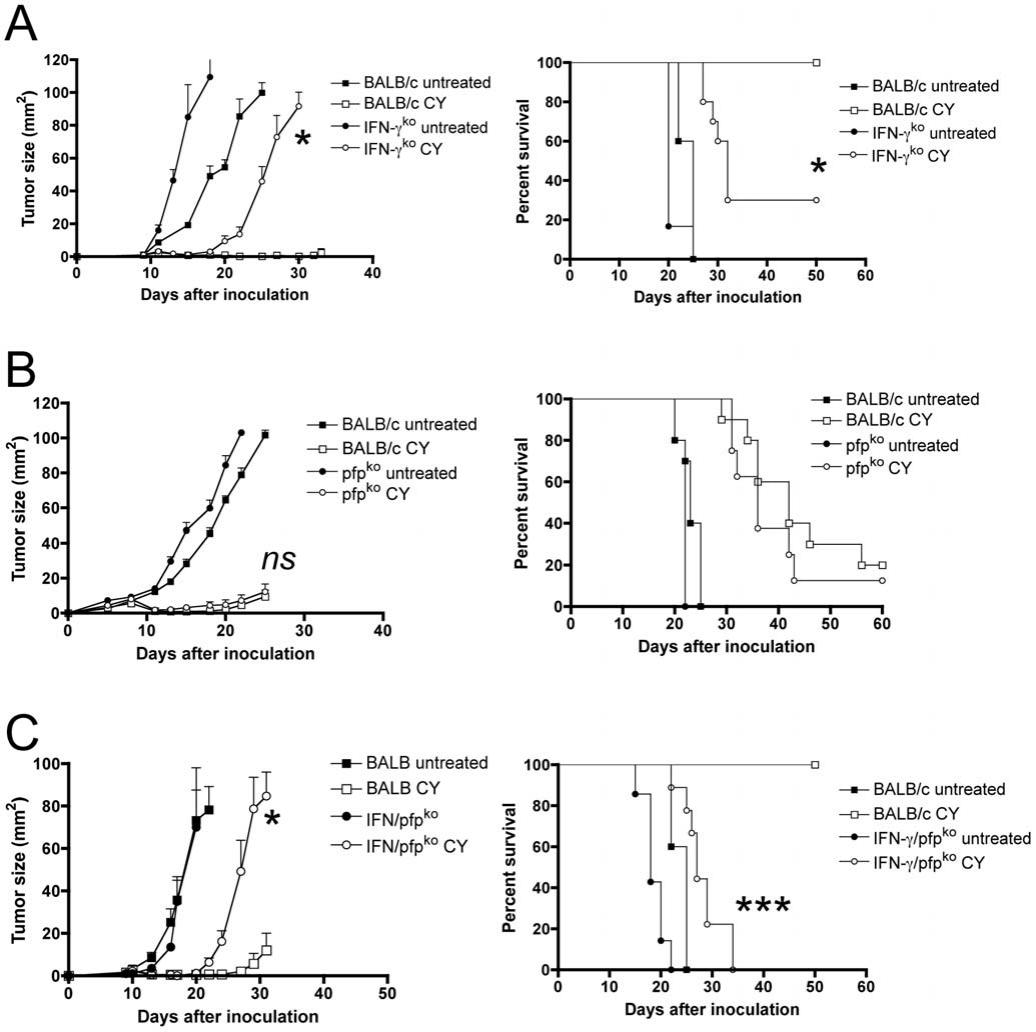
CD8 T cell effector mechanisms. (A) Tumor growth and Kaplan-Meier survival curves in CY-treated IFN-γ-deficient mice and control mice. Data shown are mean±SEM (*n* = 5) from one experiment (growth curve, left panel) or total data from two experiments (survival curve, right panel). Tumor cells were inoculated at day 0, treated with CY at day 9. * P<0.05 when CY in immunocompetent mice and IFN-γ deficient mice is compared. (B) Tumor growth curves and Kaplan-Meier survival curves in CY-treated perforin-deficient and normal control mice. *ns* = not significant when CY in immuno-competent and perforin-deficient mice are compared. Tumor cells were inoculated at day 0 and treated with CY at day 8. *ns*, not significant. (C) Tumor growth and Kaplan-Meier survival curves after CY treatment in perforin/IFN-γ double-deficient mice, compared to immunocompetent mice. Data shown are mean±SEM (*n* = 5) from one experiment (growth curve, left panel) or total data from two experiments (survival curve, right panel). Tumor cells were inoculated at day 0 and treated with CY at day 9. * P<0.05, *** P<0.001 when BALB/c *+* CY is compared with IFN-γ/*pfp*-ko + CY.

**Figure 6:**
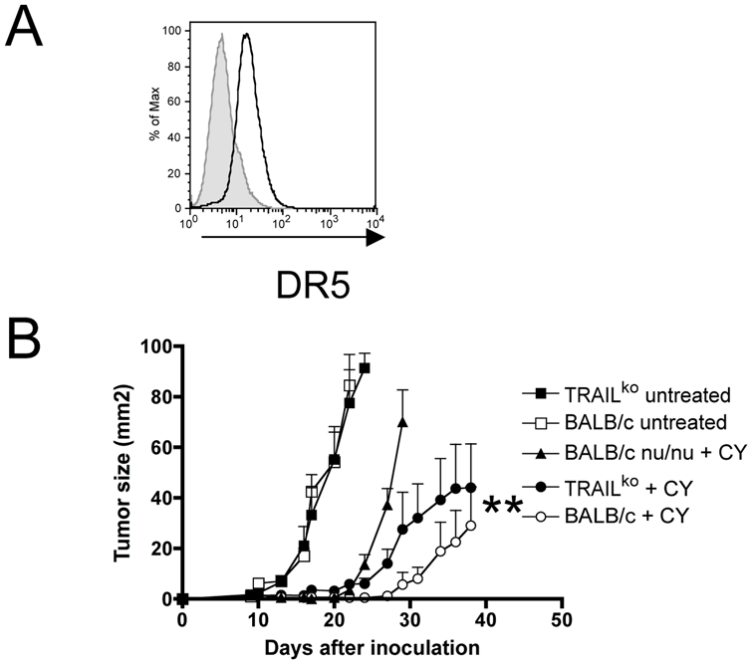
Role of TRAIL-mediated tumor cell apoptosis. (A) DR5 expression on AB1-HA tumor cells *in vitro*. (B) Tumor growth curves in CY-treated and untreated TRAIL-deficient mice, compared with immunocompetent mice and nude mice. Data shown are mean±SEM (*n* = 10) from two combined experiments. Tumor cells were inoculated at day 0 and treated with CY at day 10. ** P<0.005 when CY in BALB/c is compared with CY in TRAIL-deficient mice.

To address the question how CD8 T cells can be the key mediators for tumor resolution under conditions that limit T cell proliferation, we set out to delineate the effector mechanisms responsible for the CY-induced anti-tumor immune responses. We evaluated the roles of different CD8 T cell effector mechanisms using gene-targeted mice lacking specific CD8 T cell effector functions, i.e., IFN-γ and/or perforin. To define the role of IFN-γ, we analyzed tumor growth in IFN-γ knock-out mice and found that IFN-γ deficiency prevented the anti-tumor response in 7/10 mice (total for two experiments) (**Figure 5A**) (P<0.05 in the growth curve and P<0.05 in the survival curve). To determine whether direct CD8 T cell cytotoxicity was required for the CY-induced anti-tumor response, we assessed the efficacy of CY in perforin (*pfp*)-deficient mice. Perforin-deficiency did not abolish the anti-tumor efficacy of CY (**Figure 5B**), and there was no significant difference in survival (**Figure 5B**). It is important to note that slightly larger tumors were treated in these experiments (4–6 mm^2^ instead of 1 mm^2^ in other experiments), which has a negative impact on the efficacy of CY. This explains why CY is less efficacious in these experiments. Finally, we evaluated the efficacy of CY in IFN-γ/perforin double knock-out mice and found that combined deficiency of IFN-γ and perforin completely abrogated the CY-induced anti-tumor effects (9/9 mice, two experiments), consistent with the results from the IFN-γ-knock-out mice and highlighting the key role of this effector molecule (**Figure 5C**). In fact, these tumors grew with similar kinetics as in T-cell deficient nude mice (**Figure 6B**), consistent with our previous data [18].

### Role of TRAIL suggests a tumor cell sensitization model

As several chemotherapeutic drugs sensitize tumor cells for death ligand-mediated cell apoptosis (TRAIL) [34], we analyzed expression of the TRAIL receptor DR5 on AB1-HA tumor cells. We could detect expression of DR5 on tumor cells (**Figure 6A**) suggesting that TRAIL-mediated apoptosis could be involved in the anti-tumor effects of CY. To evaluate the *in vivo* relevance of TRAIL-mediated killing, tumor-bearing TRAIL-deficient mice were treated with CY and tumor growth in these mice was compared with immunocompetent mice. Analysis of tumor growth curves revealed that TRAIL-deficiency indeed affected the anti-tumor efficacy of CY (**Figure 6B**, P<0.005), suggesting that TRAIL could be an important effector molecule for anti-tumor CD8 T cells. Athymic nude mice were used as a control to assess the tumor growth rate in the absence of T cells.

### Agonistic anti-DR5 antibodies rescue anti-tumor efficacy in nude mice

**Figure 7:**
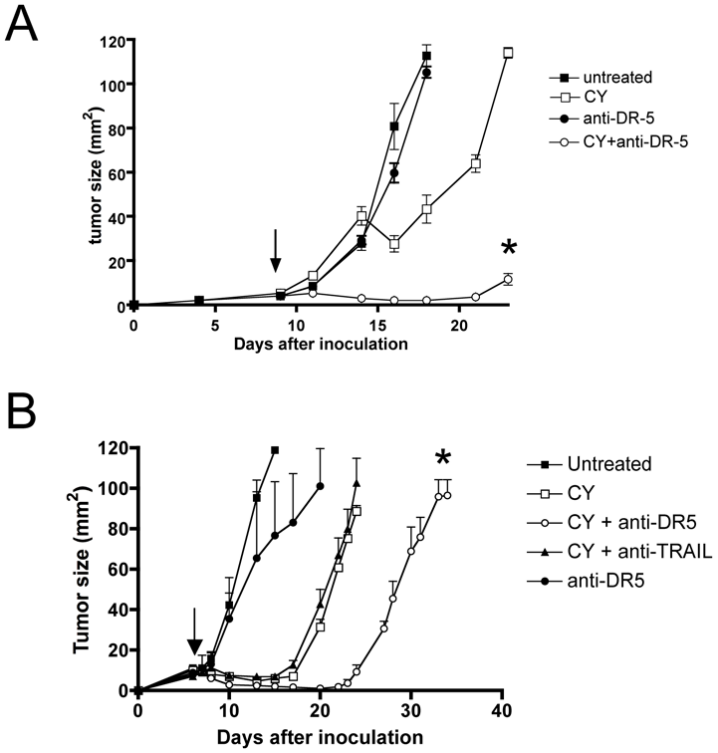
Rescue of CY anti-tumor efficacy in nude mice by anti-DR5 antibodies. Tumor-bearing athymic nude mice (inoculated at day 0) were treated with CY or with PBS at day 9 (a) or day 7 (b) after tumor cell inoculation and were treated with anti-DR5 antibody (clone MD5-1) or PBS at day 8, 12 and 16 after tumor cell inoculation. Data shown are mean±SEM (*n* = 5/group for each experiment). * P<0.05 when CY is compared with CY + anti-DR5. The effect of anti-TRAIL Ab without CY is shown in (b). Two mice were disqualified from the graph as they were found dead early in the experiment.

A TRAIL-dependent tumor-resolution mechanism predicts that the partial anti-tumor response of CY in athymic nude mice (**Figure 6B**) [18] could potentially be rescued by mimicking TRAIL-mediated tumor cell apoptosis. We tested this by combining CY-treatment in nude mice with agonistic anti-DR5 antibodies (clone MD5-1) [35], [36], reasoning that CY-sensitized tumor cells would then be killed by antibody-mediated DR5 ligation, compensating for the lack of T cell derived TRAIL. Thus, nude mice received a single dose of CY at day 9 post tumor cell inoculation (tumor size 4–6 mm^2^) and were treated with anti-DR5 on days 8, 12 and 16 post tumor cell inoculation. The results clearly show that DR5-treatment alone was completely ineffective and that CY alone had a very transient effect (**Figure 7A**). Note that the single CY treatment in nude mice was suboptimal when compared to earlier data (**Figure 6B**) [18], which can be explained by the fact that these experiments were done with CY injection at day 9 rather than day 7. Indeed, the CY efficacy in athymic nude mice is very limited when larger (>1×1 mm) tumors are treated, emphasizing the role of T cells. Indeed, repetition of the experiment with CY-treatment done at day 7 and anti-DR5 on days 6, 10 and 14 (Figure 7B) confirmed the benefit of anti-DR5 antibodies in combination with CY and also increased efficcy of earlier CY treatment (day 7 instead of 9). Combined, the data clearly show that combination of CY with the agonistic anti-DR5 antibody led to prolonged control of tumor growth (**Figure 7**). Unfortunately, the CY/anti-DR5 combination treatment was also associated with liver toxicity, preventing us from studying long-term control of tumor growth [37]. TRAIL blockade alone did not significantly affect the efficacy of CY in nude mice, suggesting that the transient anti-tumor effect did not depend on this mechanism (Figure 7B).

## Discussion

The objective of the current study was to address the chemo/immunotherapy paradox: how can CD8 T cells eradicate the tumor when the chemotherapeutic drug antagonizes their proliferation? We used CY in our AB1-HA malignant mesothelioma model to address this issue because CY eliminates AB1-HA tumors in a CD8 T cell-dependent fashion but depletes cycling T cells at the same time. Our study makes four points. First, the immune system (i.e., CD8 T cells and NK cells) is essential to achieve curative responses after chemotherapy. This could suggest that T cells and NK cells are required to prevent a small number of chemotherapy-surviving tumor cells from re-establishing a new tumor [38]. Second, the CY analogue maphosphamide induces apoptosis in tumor cells, consistent with its direct cytotoxic effects on AB1-HA tumor cells [18]. *In vivo*, the result of chemotherapy is a systemic and long-lived type-I IFN response. Third, CY depleted cycling Ki-67^hi^ T cells, blocked proliferation of CFSE-labeled tumor-specific CD8 T cells and did not expand the endogenous tumor-specific CD8 T cell response. Thus, the anti-tumor CD8 response after CY injection does not depend on proliferation for its mode of action. Fourth, multiple effector functions were important for tumor resolution: IFN-γ and TRAIL. The role of TRAIL suggests that CY sensitizes tumor cells for TRAIL-mediated apoptosis. Thus, it is possible that anti-tumor CD8 T cells use IFN-γ and TRAIL to kill tumor cells. An intriguing possibility is that TRAIL-mediated tumor cell killing contributes to immunogenic cell death.

CY kills cells by inducing apoptosis and this seems to result in a systemic type-I IFN response, confirming earlier studies [17], [29], [39] and consistent with the notion of ‘immunogenic cell death’. Similar data have been reported by Schiavoni and coworkers who used expression of Ly6C on T cells (similar to Ly6AE) and PCR to detect type-I IFN production [17]. Thus, we now confirm and extend these observations. There may be two possible sources for IFN production: the tumor cells themselves [29] and the monocytes that phagocytose apoptotic tumor cells. There is direct evidence for the former hypothesis [29], but the fact that cells exposed to the CY-related drug melphalan activate phagocytosing DCs [40] indicates that the latter may also occur. Irrespective of the mechanism, type-I IFNs are essential to generate *de novo* CD8 T cell responses to apoptotic cells [8] and to cross-presented antigens [30] and we hypothesized that CY-associated IFN responses might augment potential anti-tumor CD8 T cell responses. Indeed, this was the case in a MOPC-315 tumor model in which CY induced type-I IFN-dependent tumor regression [39]. However, CY-induced anti-tumor responses in our model are clearly less dependent on type-I IFNs, in contrast to the anti-tumor effects of poly-I:C and imiquimod in the same model [5], [23]. This indicates that CY mobilizes anti-tumor CD8 T cells in a way that does not involve type-I IFN dependent activation and/or expansion [30], [41].

CY depletes proliferating CD8 T cells, and, as we have recently shown, also CD4 T cells and foxp3+ regulatory CD4 T cells [18]. It is not entirely clear how CY has such a strong effect on proliferating T cells, although several studies suggest that it may be linked to increased numbers of NO-producing myeloid-derived suppressor cells [42], [43]. Whereas depletion of proliferating regulatory T cell is beneficial [18], [19], [44], the impact of CY on the CD8 T cell response could be negative and seems inconsistent with the key role of CD8 T cells in tumor resolution. Our explanation is that the importance of CY-triggered CD8 T cell responses lies not so much in their total numbers, but rather in their capacity to kill tumor cells. In this model, CY kills the majority of tumor cells and sensitizes the remaining tumor cells for T cell-mediated apoptosis, allowing the tumor-specific CD8 T cell pool to become effective without the need for further expansion. The benefit of regulatory T cell depletion [18], [19], [45], [46] may be that existing anti-tumor CD8 T cells are liberated from suppression, allowing them to kill the sensitized tumor cells.

A tumor cell sensitization model [34] is supported by the key role of TRAIL. Since no curative responses were ever observed in athymic nude mice, it is evident that CY does not kill all tumor cells. Therefore we propose that a subset of tumor cells that receives a sublethal hit from the drug is sensitized for apoptosis by agonistic anti-TRAIL-R (DR5) antibodies or tumor-specific CD8 T cells and most likely also NK cells. TRAIL sensitization of tumor cells, including human mesothelioma cells, by chemotherapeutic drugs has been well characterized [47], [48] and has been translated into therapeutic approaches involving soluble TRAIL or other TRAIL-receptor agonists [35], [49], [50]. It has been proposed that the synergy of TRAIL-ligation and chemotherapy can be explained by integration of the intrinsic (through chemotherapy) and alternative (death receptor) pathways of apoptosis [47]. Recently, Johnstone and colleagues showed that the histone diacetylase inhibitor vorinostat augmented the anti-tumor effects of anti-DR5 antibodies *in vivo* and *in vitro* through downregulation of the apoptosis inhibitor cellular-FLIP [51]. Similar *in vitro* data were shown earlier with cycloheximide and sensitivity to TRAIL apoptosis [52]. TRAIL sensitivity can also be increased through the proteasome inhibitor bortezomib [53]. The liver toxicity that we observed in our current study seems consistent with recent work showing that the drug 5-azacytidine sensitizes hepatocytes for TRAIL apoptosis [54] and with a recent study showing that DR5-mediated apoptosis of cholangiocytes contributes to liver disease [37].

Increased TRAIL sensitivity explains why massive T cell expansion is not necessary and why type-I IFNs are not required for the anti-tumor effect in our model. Our data suggest that CD8 T cells are a major source of TRAIL, since these cells are essential for tumor resolution. NK cells may also be required [55]. A more precise determination of the relative roles of NK cells and CD8 T cells and of the source(s) of TRAIL will be the subject of further studies, which should include measuring the in vitro sensitivity of maphosphamide-treated tumor cells to DR5 ligation. The role of IFN-γ could be related to TRAIL, since both type-I and type-II IFNs have the capacity to induce TRAIL expression [56], [57] and anti-tumor function [58]. The importance of TRAIL suggests that TRAIL production by T cells or NK cells could be an important readout to predict the efficacy of cancer vaccines or immunotherapies, possibly equally important as perforin-mediated cell apoptosis and IFN-γ.

## Materials and Methods

### Ethical statement

Animal experimentation was conducted according to University of Western Australia Animal Ethics Committee approvals and the NH&MRC code of conduct.

### Reagents and antibodies

Cyclophosphamide, maphosphamide and gemcitabine were from Sigma-Aldrich, Baxter Oncology (Halle, Germany) and the Sir Charles Gairdner Hospital Pharmacy, respectively. FLIVO caspase staining kits were from Immunochemistry Technologies and used according to the instructions. Antibodies were from BD Biosciences, eBioscience and Caltag: TCRβ-AF488 (H57-597), CD3ε-FITC (145-2C11), CD4-PE/CD4-PECy7 (RM4-5), CD8-PECy5/CD8-APC-AF750 (53–6.7), CD19-PECy7 (eBio1D3), Ly6A/E-FITC (Sca1), Ki-67-FITC (B56), CD43-FITC (1B11) and DR5-PE (MD5-1). Flow cytometry was performed on FACSCalibur and FACSCanto II instruments and analyzed using Flowjo (TreeStar).

### Mice

Female BALB/c (H-2^d^) wild-type and nude mice (6–8 wk old) were purchased from the Animal Resources Centre (Canning Vale, Australia). TCR transgenic CL4 mice, expressing a TCR specific for the H-2^d^-restricted peptide IYSTVASSL (residues 518–526) from the influenza virus A/PR8/8/34 HA protein, were generated and screened as described [15]. TRAIL-deficient mice were originally generated at Immunex Corporation [35], [59] and were backcrossed to BALB/c at the Peter MacCallum Cancer Centre (Melbourne, Australia) (n = 12). Perforin-IFN-γ double-deficient mice were bred at the Peter MacCallum Cancer Centre. BALB/c perforin-deficient mice were originally generated at the Peter MacCallum Cancer Centre [60] and bred at the Animal Care Unit at UWA (Perth, Australia).

### Tumor cell culture and inoculation

Generation and maintenance of the BALB/c-derived mouse mesothelioma cell line AB1 and transfection with the HA gene (AB1-HA) has been described [15], [16]. AB1-HA cells (1×10^6^ in PBS) were injected s.c. into the right flank of recipient mice and tumor growth monitored using microcalipers. Mice were euthanized when tumors reached 10×10 mm as per Animal Ethics guidelines.

### Pentamer staining

For pentamer (ProImmune) staining, cells were blocked with 20 µl FCS (10′, RT) after which 5 µl pentamer was added (30′, 4°C). Cells were stained with mAbs against CD8 and TCRβ and anti-CD19 were included to reduce background.

### Lyons-Parish analysis of Ag presentation

For CFSE (Molecular Probes) labeling, lymph node cells from TCR-transgenic CL4 mice were incubated with 2.5 µM CFSE (10′ RT) and then centrifuged through a FCS cushion. A total of 1×10^7^ cells were injected i.v. into recipient mice. CFSE-labeled cells were recovered 7 days after adoptive transfer and analyzed by FACS.

### In vivo antibody treatments

The IFN-α/β neutralizing sheep Ig and matching normal sheep Ig [17], [61] were used as described [5]. Mice were i.v. injected with 0.2 ml of immunoglobulins on day –1, +2, and +4 with respect to CY administration. Monoclonal anti-IFN-γ antibodies (clone XMG 1.2) were purified from hybridoma supernatant and were injected from day 7 after tumor cell inoculation onwards (q3dx4, 100 µg per injection). CD8α T cell depletion was performed using purified YTS.169 monoclonal antibody (Dr Kathy Davern, Monoclonal Antibody Facility, Western Australian Institute for Medical Research) as previously described [5]. CD8 depletion (>95%) was verified by FACS analysis of PBL. Agonistic anti-DR5 antibody (clone MD5-1) [36] was injected i.p. at days -1, 3 and 7 relative to CY treatment (50 µg/injection). Neutralizing TRAIL antibodies (clone N2B2) [36] were injected i.p. at days −1, 2, 5 and 8 relative to CY treatment (200 µg/injection).

### Statistics

Data were statistically evaluated using Prism software (GraphPad). Survival responses were analyzed by Kaplan-Meyer using log-rank test. Growth curves were compared using a two-tailed paired t test, with pairs defined by time point. Other variables were compared using a two-tailed unpaired t test as indicated. Survival curves were compared using the log rank test. Significance was defined as p<0.05 and is indicated in the figures and figure legends by asterisks.
